# Algebra dissociates from arithmetic in the brain semantic network

**DOI:** 10.1186/s12993-022-00186-4

**Published:** 2022-01-07

**Authors:** Dazhi Cheng, Mengyi Li, Jiaxin Cui, Li Wang, Naiyi Wang, Liangyuan Ouyang, Xiaozhuang Wang, Xuejun Bai, Xinlin Zhou

**Affiliations:** 1 State Key Laboratory of Cognitive Neuroscience and Learning, Institute of Cognitive Neuroscience and Learning, IDG/McGovern Institute for Brain Research, Beijing Normal University, No.19, Xinjiekouwai Street, Haidian District, Beijing, 100875 China; 2 Lab for Educational Neuroscience, Center for Educational Science and Technology, Faculty of Education, Beijing Normal University, Beijing, 100875 China; 3 Advanced Innovation Center for Future Education, Beijing Normal University, Beijing, 100875 China; 4 Department of Pediatric Neurology, Capital Institute of Pediatrics, Beijing, 100020 China; 5 College of Education, Hebei Normal University, Shijiazhuang, 050024 China; 6 Faculty of Psychology, Tianjin Normal University, Tianjin, 300387 China

**Keywords:** Algebra, Arithmetic, Semantic network, Phonological network, Visuospatial network

## Abstract

**Background:**

Mathematical expressions mainly include arithmetic (such as 8 − (1 + 3)) and algebra (such as a − (b + c)). Previous studies have shown that both algebraic processing and arithmetic involved the bilateral parietal brain regions. Although previous studies have revealed that algebra was dissociated from arithmetic, the neural bases of the dissociation between algebraic processing and arithmetic is still unclear. The present study uses functional magnetic resonance imaging (fMRI) to identify the specific brain networks for algebraic and arithmetic processing.

**Methods:**

Using fMRI, this study scanned 30 undergraduates and directly compared the brain activation during algebra and arithmetic. Brain activations, single-trial (item-wise) interindividual correlation and mean-trial interindividual correlation related to algebra processing were compared with those related to arithmetic. The functional connectivity was analyzed by a seed-based region of interest (ROI)-to-ROI analysis.

**Results:**

Brain activation analyses showed that algebra elicited greater activation in the angular gyrus and arithmetic elicited greater activation in the bilateral supplementary motor area, left insula, and left inferior parietal lobule. Interindividual single-trial brain-behavior correlation revealed significant brain-behavior correlations in the semantic network, including the middle temporal gyri, inferior frontal gyri, dorsomedial prefrontal cortices, and left angular gyrus, for algebra. For arithmetic, the significant brain-behavior correlations were located in the phonological network, including the precentral gyrus and supplementary motor area, and in the visuospatial network, including the bilateral superior parietal lobules. For algebra, significant positive functional connectivity was observed between the visuospatial network and semantic network, whereas for arithmetic, significant positive functional connectivity was observed only between the visuospatial network and phonological network.

**Conclusion:**

These findings suggest that algebra relies on the semantic network and conversely, arithmetic relies on the phonological and visuospatial networks.

## Background

Mathematical expressions are frequently conceived and represented in arithmetic and algebra. Arithmetic is concretely represented as the combination of digits and operators (such as 8−(1 + 3)). Contrastingly, algebra includes abstract operations with letters and operators (such as a−(b + c)) and has more abstract representation than arithmetic in form [1]. Although previous studies demonstrated the dissociation between algebra and arithmetic [2–8], the neural mechanisms underlying this dissociation remain unclear. The current study used functional magnetic resonance imaging (fMRI) to examine how algebraic processing dissociates from arithmetic processing in brain networks.

### Arithmetic in the brain

In the arithmetic expression “4 × 5−9,” the sub-expression “4 × 5” is recursively combined with “9” by subtraction. It has been proposed that the arithmetic operations both involved numerical quantity calculation and order-rule of arithmetic operations [9]. Neuroimaging and neuropsychological studies have revealed that arithmetic processing is subserved by the visuospatial network, which typically includes the bilateral intraparietal sulcus (IPS) and bilateral inferior and superior parietal lobules [10–14]. Several studies have shown that arithmetic activated the bilateral parietal lobules also support number processing [14–17]. Approximately 73% of the behavioral variance of visuospatial magnitude processing has been attributed to the change in cortical thickness in the right superior parietal cortex, and approximately 55% of the behavioral variance of children’s arithmetic abilities has been attributed to the change in cortical folding in the right IPS [18]. Patients with injuries in the parietal cortex typically experience arithmetic impairment [19, 20] and impaired visuospatial performance [21].

During arithmetic computation, the phonological system also supports the coding of visual or auditory numbers, the results of numerical calculations were temporarily stored in the phonological loop of working memory [22, 23]. fMRI studies showed that arithmetic processing relies on the phonological network, which typically includes the bilateral supplementary motor areas and the precentral gyrus [15, 24–28]. For example, Zhou et al. [27] reported that both addition and multiplication are supported by a broad neural system involving the supplementary motor areas, precentral gyrus, bilateral IPS, and middle frontal gyrus. One recent study showed that exact arithmetic involves more phonological or verbal processing localized in the left Rolandic operculum, precentral gyrus, and supplementary motor area than approximate arithmetic [25]. Neuropsychological investigations have also shown that patients with infarction in the left frontal lobe (including the precentral gyrus) have difficulty retrieving arithmetic facts [29, 30].

### Algebra in the brain

Algebra (such as a−(b + c)) contains abstract operations with letters and operators, which is similar to arithmetic in procedural knowledge [9, 31]. Algebra is similar to arithmetic in form, but the former involves more abstract representation than the latter. Evidence from several domains of cognitive neuroscience has illustrated the association and dissociation between the processing of algebra and arithmetic.

Both algebra and arithmetic processing is associated with regions in the parietal lobes. As mentioned above, arithmetic processing typically activate the bilateral IPS [10–14, 28]. Similarly, compared to language syntax processing, algebraic processing typically recruits the bilateral parietal brain regions around the IPS more, to represent numerical magnitude [32–34]. It dissociated from the typical brain activation in classic language processing including the left inferior frontal gyrus and posterior and anterior middle temporal gyrus [12, 13, 35, 36]. Algebraic operations did not recruit any more language resources than did simple reading (i.e., the grammar task); in contrast, they did rely on areas previously linked to arithmetic cognition.

Algebraic processing involves more abstract language symbolic; thus, it dissociated from arithmetic in the application of conceptual and semantic knowledge [12, 32, 37, 38]. For example, Geary et al. [38] found that algebraic cognition was related to semantic memory systems of addition facts in ninth graders. Neuroimaging studies further implied that the brain network’s response to algebra is also associated with language semantic processing. A meta-analysis study of 120 functional neuroimaging studies suggested that the semantic processing system actually covers a wide range of brain regions including seven left-lateralized regions: posterior inferior parietal lobe (angular gyrus), middle temporal gyrus, fusiform and parahippocampal gyri, dorsomedial prefrontal cortex, inferior frontal gyrus, ventromedial prefrontal cortex, and posterior cingulate gyrus [39]. In a task involving algebraic transformation using non-arithmetic symbols (“artificial algebra”), adults showed activation in the left posterior parietal and left prefrontal regions [37]. When both expert mathematicians and nonmathematicians made semantic judgements regarding algebraic statements, a bilateral network involving the prefrontal, parietal, and inferior temporal regions was recruited [32]. Recent studies have shown that compared to simple numerical processing and arithmetical computation, algebraic terms elicited greater activation in the typically semantic network such as left angular gyrus, left middle temporal gyrus, and left inferior frontal gyrus [12, 40]. These regions typically overlap with the typically semantic network [39]. These findings suggested that algebra is dissociated from arithmetic in brain activation.

### The present study

Although previous studies demonstrated the dissociation between algebra and arithmetic [2, 4–8, 12, 32, 35, 37, 38], the neural bases of the dissociation between algebraic processing and arithmetic is still unclear. This study uses fMRI to identify the specific neural bases for the dissociation between algebraic and arithmetic processing. The arithmetic and algebraic tasks used in this study were matched in surface form and difficulty. In the present study, algebraic used the expressions of the operational principles for addition, subtraction, multiplication, and division (such as a−(b + c)). To match the algebraic task, we used the expressions requiring arithmetic procedures (such as 8−(1 + 3)) rather than that requiring the simple retrieval of arithmetic facts. For each problem of the algebraic and arithmetic tasks, two algebraic expressions or two arithmetic expressions were presented simultaneously on the screen, one at the top and one at the bottom. Participants were asked to judge whether the results of the two expressions were equal.

Prior studies have indicated that arithmetic involves numerical quantity operations and phonological processing [10–14, 28]. Contrastingly, the application of semantic knowledge is involved in algebraic processing [12]. Therefore, we hypothesized that algebra relies more on the semantic network and arithmetic involves the phonological and visuospatial networks based on the aforementioned reports. It is important to clarify whether algebra relies on visuospatial or semantic networks. If algebra and arithmetic rely on different brain networks, it has strong implications for education and teaching. Distinct approaches should be used to learn algebra and arithmetic. A verbalization approach emphasizing semantic comprehension was used to learn algebra, and visualization and phonological approaches were used to learn arithmetic [41]. To test this hypothesis, we compared the fMRI data regarding brain activation for algebra with that for arithmetic, and we conducted a single-trial (item-wise) interindividual correlation analysis and a traditional mean-trial interindividual correlation analysis to examine the brain-behavior correlations [25, 42].

## Methods

### Participants

Thirty right-handed undergraduate students (15 male and 15 female; mean age = 21.92 years, range: 18–25 years) were recruited to participate in this study from Beijing Normal University, China. They majored in a wide range of disciplines except for mathematics. These participants reported having no history of neurological disorders or head injuries. The experiment was fully explained to the participants before informed consent was obtained. This study was approved by the Institutional Review Board of the State Key Laboratory of Cognitive Neuroscience and Learning at Beijing Normal University.

### Materials

**Fig. 1:**
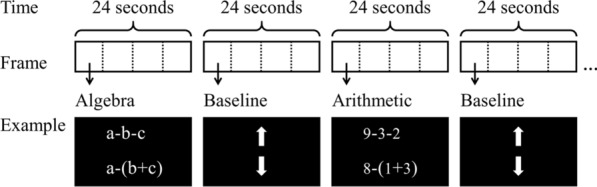
Experimental procedure and examples of trials. Each block consisted of four trials lasting 24 s. For each trial in the experimental blocks, two expressions were presented synchronously on the screen. Participants were asked to judge whether the two expressions were equal. For each trial in the baseline blocks, two arrows were presented synchronously on the screen. Participants were asked to judge whether the two arrows pointed in the same direction

Each problem of the algebraic and arithmetic tasks included two expressions of the two-step operation for addition, subtraction, multiplication, and division. Two expressions were presented synchronously in white against a black background (with a red, green, and blue value of 0, 0, 0), one at the top of the screen and one at the bottom of the screen (Fig. 1). Participants were asked to judge whether the results of the two expressions were equal. The problems of the algebraic task were presented in lowercase letters (i.e., a, b, c) and operators (i.e., + , −, × , ÷). All equal problems were selected from the operational principles acquired by students in primary school, mainly including the associative law, commutative law, and distributive law. Because there were few two-step operational principles and the difficulty level need to be matched, the algebraic task only contained 16 problems. Half of the problems were equal problems, and each expression has been used in one equal problem and one unequal problem. The previous fMRI studies of mathematical processing have suggested that using 16 trials or less can yield stable and reliable results [40, 43]. To match the algebraic task, we designed the same number of problems for the arithmetic task. All problems were presented in digits (i.e., 1–9) and operators (i.e., + , −, × , ÷), which required judging whether two expressions were equal by numerical calculation. The average length of the problems was controlled between two tasks, and all problems of the algebraic and arithmetic tasks can be seen in Appendix.

The stimulus presentation and behavioral data recordings were programmed using E-prime software (Version 1.1, Psychology Software Tools, Inc., www.pstnet.com) on a Pentium 4 laptop. Stimuli were projected onto a translucent screen placed at the back of the magnet bore. Participants viewed the screen through a mirror mounted on the head coil at 30 cm from the eyes.

### Procedure

Prior to scanning, the participants underwent a training session with the same type of materials as a formal experiment to ensure that they understood the instructions of this experiment. After that, participants were required to complete the experimental tasks in the scanner. The experiment used a block design because it has high efficiency, which can increase the statistical power than event-related design of similar timing [44–46].

The scanning session was organized into two runs, each lasting 192 s. Each run consisted of four experimental blocks (two experimental blocks for each task) and four baseline blocks (Fig. 1). Thus, there were a total of four blocks for each condition. Each block with four trials lasted 24 s, and each trial lasted 6 s. The balanced Latin square design [47] was used to counterbalance the order effect of the two experimental tasks, and the presentation order of trials was random. There was a 1 min rest period after each run.

For each trial in the experimental blocks, two expressions were presented synchronously on the screen, one at the top and one at the bottom. Participants were asked to judge whether the two expressions were equal. For each trial in the baseline blocks, two arrows were presented synchronously on the screen, one at the top and one at the bottom. Participants were asked to judge whether the two arrows pointed in the same direction. The arrow judgement was the baseline to remove consistent or inconsistent judgements and the left or right finger motor response, which are the common judgement and response patterns across all the conditions. Each trial lasted 6 s, and a fixation cross was displayed to fill the remaining time if the participant did not use all 6 s. Half of the participants responded to the trials by pressing a key on a response box on their left with their left index finger when the two mathematical expressions or arrows were equal. The remaining participants responded to the trials by pressing a key on a response box on their right with their right index finger. Both accuracy and speed were emphasized.

### fMRI data acquisition

Images were obtained using a Siemens (Munich, Germany) 3T Trio MRI scanner using a standard eight-channel head coil. After automatic shimming of the magnetic field, three-dimensional high-resolution T1 anatomical images were acquired for co-registration with the functional images. Next, functional volumes were acquired using a multiple slice T2*-weighted echo planar imaging (EPI) sequence with the following parameters: in-plane resolution = 3.125 × 3.125 mm^2^; repetition time = 2000 ms; echo time = 30 ms; flip angle = 90°; field of view = 240 × 240 mm^2^; matrix dimensions = 64 × 64; field of view = 200 mm; and slice thickness = 4 mm. The entire brain was imaged in 32 slices.

### Statistical analysis of the fMRI data

Individual MRI datasets were analyzed using SPM12 software (Wellcome Department of Imaging Neurosciences, University College London, UK; http://www.fil.ion.ucl.ac.uk/spm). All volumes were realigned to the first volume and spatially normalized to a common value to correct for whole brain differences over time. Images were then smoothed using an isotropic Gaussian kernel of 4 mm and high-pass filtered at a cut-off of 128 s.

#### Brain activation analysis

After preprocessing, parameter-estimated images for individual participants across the whole brain were calculated. Group analyses with random effects were conducted using the univariate mass analysis of variance (ANOVA) on the brain activation maps of all participants with material type as the independent variable, the separate ANOVA was calculated for each voxel of the brain map. Then, the brain activation for each type of material relative to fixation was calculated. Brain activations for the two types of materials were compared. We used the stringent threshold at cluster-level *p* < 0.05 false discovery rate (FDR)-corrected for multiple comparisons at the whole-brain level, with underlying voxel-level *p* < 0.001 uncorrected. We also reported the lenient threshold at voxel-level *p* < 0.05 uncorrected.

#### Brain-behavior correlation analysis

The brain-behavior correlation analyses are the individual differences analyses, which are different from the within-subject analyses by contrasting different experimental conditions. The within-subject analyses are most sensitive to processes that are consistent across all participants, including those that are necessary for performing a task. In contrast, individual differences analyses of the brain-behavior correlation are most sensitive to processes that can be recruited in varying degrees to produce incremental gains in performance [48]. Although there was no within-subject difference of behavioral results between algebraic and arithmetic tasks, the brain-behavior correlation analyses can also be used based on the individual differences within each task. Using the within-subject analyses by contrast brain activation of different experimental conditions and individual differences analyses by brain-behavior correlation together can help provide an accurate and representative neural mechanism of cognitive processing [48, 49].

Previous studies have proven that single-trial (item-wise) analyses also applied to the data of block design [50, 51]. Thus, we first use the single-trial (item-wise) interindividual correlation analysis that has been reported in previous fMRI and event-related potential (ERP) studies that used to examine the brain-behavior correlations [25, 42]. First, the correlation between the brain activation maps and the reaction times (RTs) was determined for each trial. Then, a one-sample *t*-test on the correlation coefficients obtained for all trials of one type of processing against zero was performed. The analysis was performed separately for algebra and arithmetic tasks. The single-trial correlation has been reported as more effective than the traditional mean-trial correlation as it can filter out much of the noise that exists after the first step [42].

The traditional mean-trial interindividual correlation was also used, and the results were compared with those of the single-trial correlation. The mean brain activation map and the mean RT for each type of processing for each participant were determined. Then, a correlation analysis between the mean brain activation map and the RT for all participants was performed. To compare the results of the single-trial and mean-trial correlation analyses, we used the stringent threshold at cluster-level *p* < 0.05 FDR-corrected with underlying voxel-level *p* < 0.001 uncorrected, and the lenient threshold at voxel-level *p* < 0.05 uncorrected simultaneously.

Further, we defined seven functional regions of interest (ROIs) based on results from previous studies for the semantic, phonological, and visuospatial networks. Based on the meta-analysis of 120 functional neuroimaging studies of semantic processing [39], we defined four ROIs of the semantic network in the left hemisphere that included the middle temporal gyrus (Montreal Neurological Institute (MNI) coordinates [−  45,  − 21,  − 15]), inferior frontal gyrus (MNI coordinates [− 51, 24, 0]), dorsomedial prefrontal cortex (MNI coordinates [− 6,  − 48, 30]), and angular gyrus (MNI coordinates [− 57,  − 45, 30]). Based on the fMRI studies of phonological processing for arithmetic [27], we defined two ROIs of the phonological network in the left hemisphere that included the precentral gyrus (MNI coordinates [− 45, 4, 30]) and supplementary motor area (MNI coordinates [− 3, 3, 58]). Based on the fMRI studies of visuospatial processing for arithmetic [52], we defined one ROI of the visuospatial network in the left superior parietal lobule (MNI coordinates [− 32, − 68, 56]). Each ROI was a sphere with a radius of 9 mm. These ROIs were used to compare the levels of brain-behavior correlations between algebra and arithmetic.

#### Functional connectivity analysis

The functional connectivity was analyzed by a seed-based ROI-to-ROI analysis in the CONN toolbox using SPM preprocessed data [53]. The ROIs were consistent with the ROIs of the brain-behavior correlation analysis, including four ROIs of the semantic network (i.e., middle temporal gyrus, inferior frontal gyrus, dorsomedial prefrontal cortex, and angular gyrus), two ROIs of the phonological network (i.e., precentral gyrus and supplementary motor area), and one ROI of the visuospatial network (i.e., superior parietal lobule) in the left hemisphere. In the first-level analyses, Pearson’s correlation coefficients were calculated between the time courses of each pair of ROIs for each subject, and these correlation coefficients were converted to normally distributed z-scores using Fisher’s transform. In the second-level analysis, the one-sample *t*-test on the z-scores against zero was performed separately for algebra and arithmetic tasks. We used the significant threshold at *p* < 0.05 FDR-corrected.

## Results

### Behavioral results

The mean RT was 2653 ms for algebraic task and 2855 ms for arithmetic task. The mean accuracy was 87.6% and 86.8%, respectively. RTs and accuracy were analyzed with a repeated measure ANOVA. The main effect of stimulus type was not significant for RTs (*F* (1, 29) = 2.55, *p* = 0.116, *η*^2^ = 0.042) or accuracy (*F* (1, 29) = 1.10, *p* = 0.754, *η*^2^ = 0.002).

### Brain activations

**Fig. 2:**
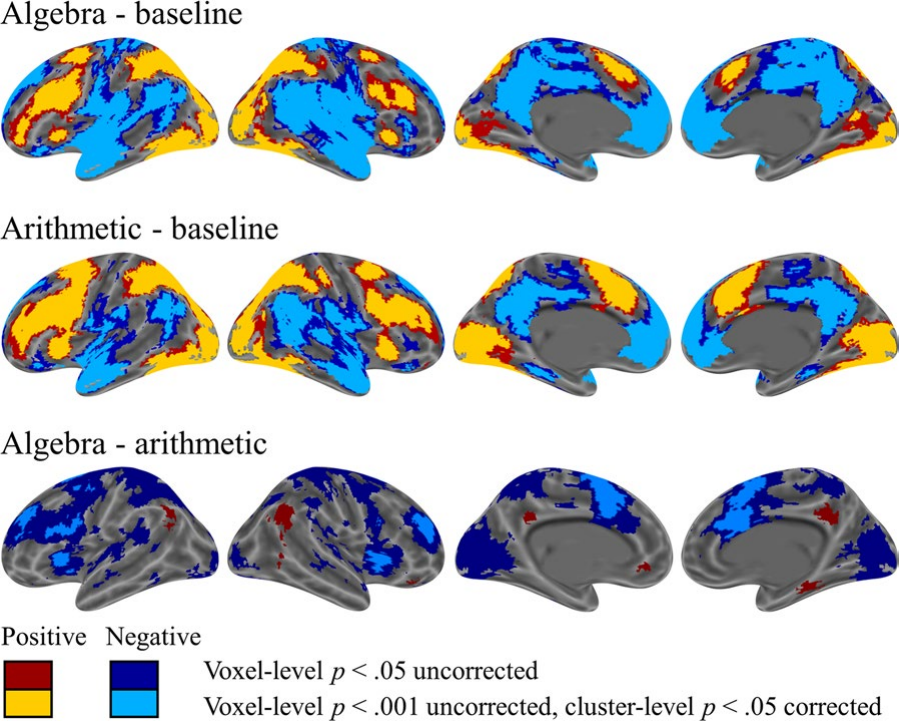
Brain activations for algebra and arithmetic relative to baseline and the contrast between algebra and arithmetic

The brain activations for algebra and arithmetic relative to baseline are displayed in Fig. 2. Algebra and arithmetic elicited significant activation in a broad array of brain regions, including the bilateral parietal, frontal, and occipital gyri. The differences in brain activation between algebra and arithmetic are shown in Fig. 2 and Table 1. Algebra had greater activation in the bilateral angular gyrus relative to arithmetic. In contrast, arithmetic elicited greater activation in the bilateral supplementary motor areas, left insula, and left inferior parietal lobule than algebra.

**Table 1:** Brain regions with significantly different activations for algebra and arithmetic

Brain region	Cluster size	*p* value (cluster-corrected)	*t* value (peak)	Coordinates		
				x	y	z
Algebra > arithmetic						
No significantly difference						
Arithmetic > algebra						
Left supplementary motor area and right middle frontal gyrus	1715	< .001	− 6.55	27	42	21
Left insula	629	< .001	− 6.08	− 33	21	9
Left lingual	358	< .001	− 5.05	− 3	− 72	6

Coordinates are in Montreal Neurological Institute space. Extent threshold: cluster-level *p* < .05 FDR-corrected with underlying voxel-level *p* < .001 uncorrected

### Brain-behavior correlations

**Fig. 3:**
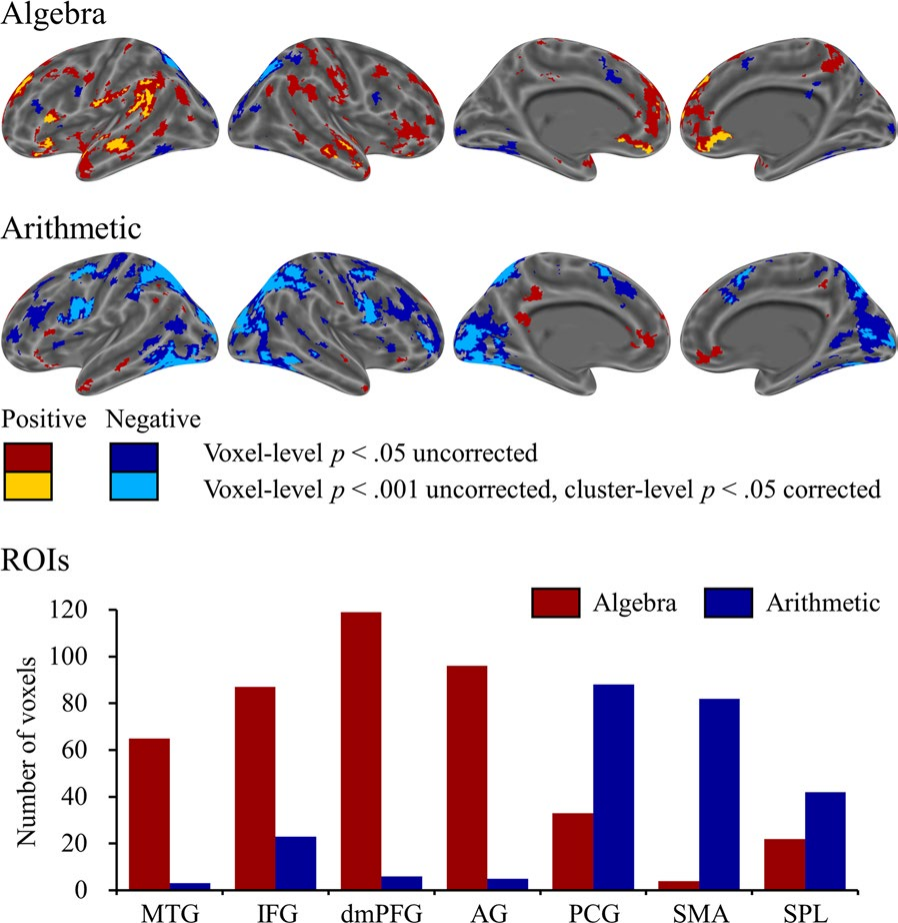
The single-trial brain-behavior correlations for algebra and arithmetic, and the number of voxels of the single-trial brain-behavior correlations for algebra and arithmetic in seven ROIs of the semantic, phonological, and visuospatial networks are shown. Semantic network: *MTG* middle temporal gyrus, *IFG* inferior frontal gyrus, *dmPFC* dorsomedial prefrontal cortex, *AG* angular gyrus. Phonologic network: *PCG* precentral gyrus, *SMA* supplementary motor area; visuospatial network: *SPL* superior parietal lobule

The single-trial brain-behavior correlations for algebra and arithmetic are displayed in Fig. 3 and Table 2. At the threshold of cluster-level *p* < 0.05 FDR-corrected with underlying voxel-level *p* < 0.001 uncorrected, significant positive brain-behavior correlations were observed in the bilateral middle temporal gyri, bilateral inferior frontal gyri, bilateral dorsomedial prefrontal cortices, and left angular gyrus for algebra. Significant negative brain-behavior correlations were observed in the bilateral superior parietal lobules for algebra. For arithmetic, significant positive brain-behavior correlations were observed in the right inferior temporal gyrus and negative brain-behavior correlations were observed in the bilateral superior parietal lobules, bilateral middle occipital gyri, bilateral precentral gyri, left supplementary motor area, left insula, and bilateral cerebella. Figure 3 shows the number of voxels of the single-trial brain-behavior correlations for algebra and arithmetic in the ROIs under the threshold of voxel-level *p* < 0.05 uncorrected. Algebra elicited a larger number of voxels in four ROIs of the semantic network, including the middle temporal gyrus, inferior frontal gyrus, dorsomedial prefrontal cortex, and angular gyrus. Arithmetic elicited a greater brain-behavior correlation and a larger number of voxels in three ROIs of the phonological and visuospatial networks, including the precentral gyrus, supplementary motor area, and superior parietal lobule.

**Table 2:** Brain regions with positive and negative brain-behavior correlations for algebra and arithmetic based on single-trial correlations

Brain region	Cluster size	*p* value (cluster-corrected)	*t* value (peak)	Coordinates		
				x	y	z
Positive correlations for algebra						
Left superior frontal gyrus	79	.008	6.38	− 21	45	39
Left middle temporal gyrus	70	.012	6.14	− 48	− 21	− 12
Left postcentral gyrus	89	.007	6.02	− 66	− 21	24
Left middle temporal gyrus	126	< .001	5.81	− 66	− 45	12
Right middle temporal gyrus	81	.007	5.73	60	− 18	− 9
Left superior medial frontal gyrus	214	< .001	5.68	0	63	12
Left inferior frontal gyrus (Triangle)	285	< .001	5.65	− 54	24	3
Negative correlations for algebra						
Right superior parietal lobule	129	< .001	− 8.33	24	− 63	42
Left superior parietal lobule	66	.013	− 7.25	− 24	− 63	45
Positive correlations for arithmetic						
No significantly correlation						
Negative correlations for arithmetic						
Left superior parietal lobule	678	< .001	− 12.74	− 27	− 63	51
Left precentral gyrus	227	< .001	− 9.79	− 51	0	27
Right middle occipital gyrus	666	< .001	− 7.91	27	− 87	15
Left middle occipital gyrus	1116	< .001	− 7.75	− 30	− 84	12
Right middle frontal gyrus	172	< .001	− 7.34	36	3	63
Right precentral gyrus	212	< .001	− 7.08	57	12	33
Left precentral gyrus	151	< .001	− 6.80	− 36	0	57
Right cerebellum (7)	103	.002	− 6.57	30	− 72	− 51
Left supplementary motor area	81	.007	− 5.96	− 6	9	57
Right fusiform	193	< .001	− 5.84	27	− 78	− 15
Right inferior temporal gyrus	72	.010	− 5.71	51	− 48	− 9
Right inferior frontal gyrus (Triangle)	64	.014	− 5.27	33	27	27
Right inferior frontal gyrus (Triangle)	111	.002	− 5.09	51	39	15

Coordinates are in Montreal Neurological Institute space. Extent threshold: cluster-level *p* < .05 FDR-corrected with underlying voxel-level *p* < .001 uncorrected

**Fig. 4:**
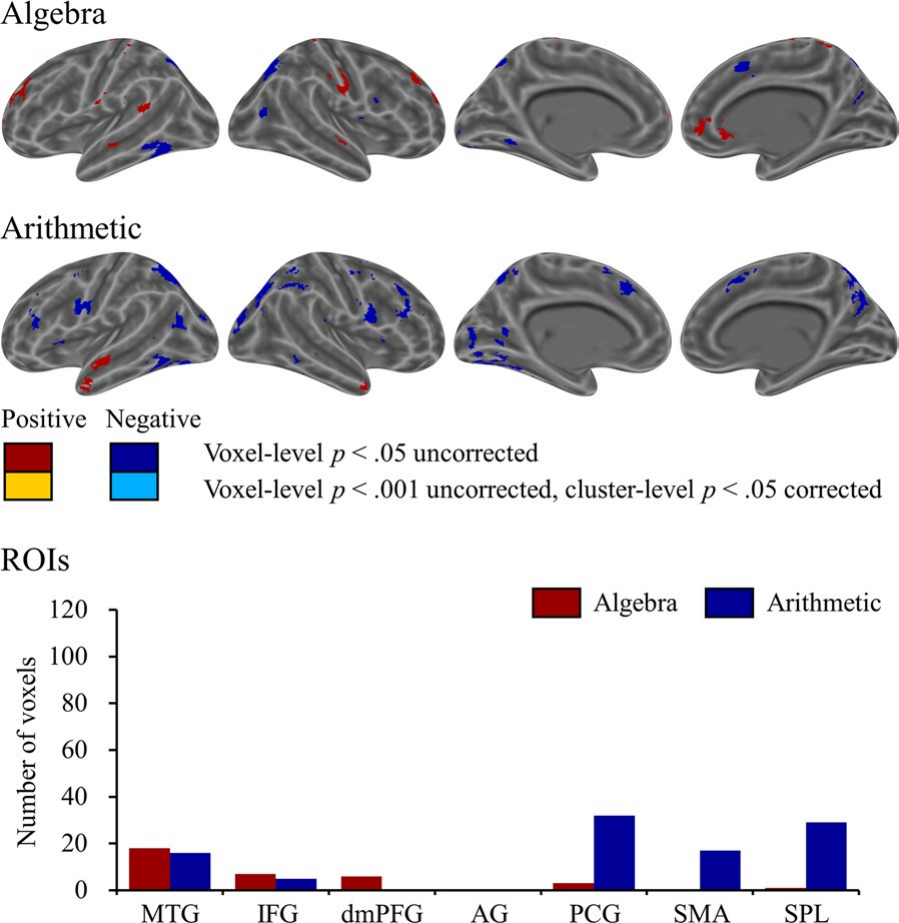
The mean-trial brain-behavior correlations for algebra and arithmetic, and the number of voxels of the mean-trial brain-behavior correlations for algebra and arithmetic in seven ROIs of the semantic, phonological, and visuospatial networks are shown. Semantic network: *MTG* middle temporal gyrus, *IFG* inferior frontal gyrus, *dmPFC* dorsomedial prefrontal cortex, *AG* angular gyrus. Phonologic network: *PCG* precentral gyrus, *SMA* supplementary motor area. Visuospatial network: *SPL* superior parietal lobule

The traditional mean-trial brain-behavior correlations for algebra and arithmetic are displayed in Fig. 4. Only at the lenient threshold of voxel-level *p* < 0.05 uncorrected, significant positive brain-behavior correlations were observed in the bilateral middle temporal gyri and bilateral dorsomedial prefrontal cortices, and negative brain-behavior correlations were observed in the bilateral superior parietal lobules for algebra. Significant positive brain-behavior correlations were observed in the bilateral inferior temporal gyri, and negative brain-behavior correlations were observed in the bilateral superior parietal lobules, bilateral middle occipital gyri, bilateral precentral gyri, and bilateral cerebella for arithmetic. Figure 4 shows the number of voxels of the mean-trial brain-behavior correlations for algebra and arithmetic for each ROI under the threshold of voxel-level *p* < 0.05 uncorrected.

### Functional connectivity

**Fig. 5:**
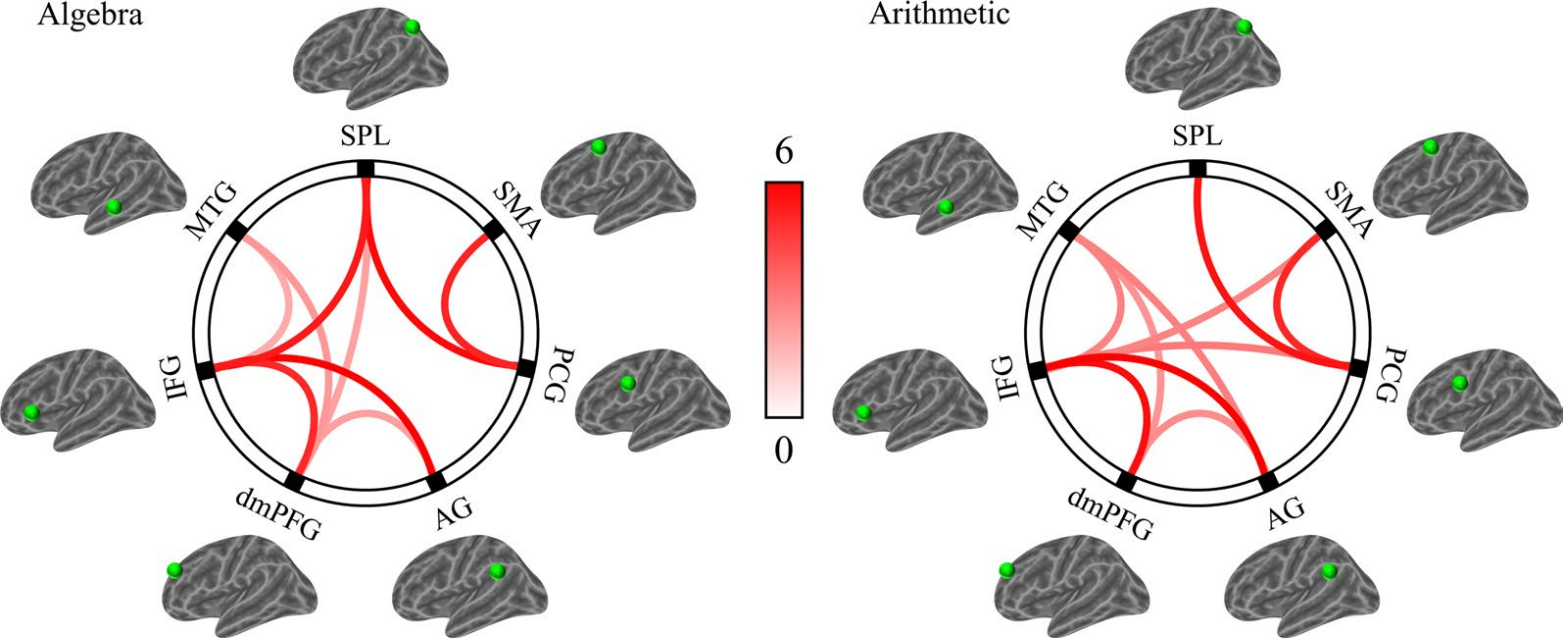
Functional connectivity between seven ROIs of the semantic, phonological, and visuospatial networks for algebra and arithmetic under the threshold of *p* < .05 FDR-corrected. Semantic network: *MTG* middle temporal gyrus, *IFG* inferior frontal gyrus, *dmPFC* dorsomedial prefrontal cortex, *AG* angular gyrus. Phonologic network: *PCG* precentral gyrus, *SMA* supplementary motor area. Visuospatial network: *SPL* superior parietal lobule

The functional connectivity between seven ROIs of the semantic, phonological, and visuospatial networks for algebra and arithmetic are shown in Fig. 5 and Table 3. At the threshold of *p* < 0.05 FDR-corrected, the positive functional connectivity within the semantic network (i.e., middle temporal gyrus, inferior frontal gyrus, dorsomedial prefrontal cortex, and angular gyrus) and phonological network (i.e., precentral gyrus and supplementary motor area) were observed for algebra and arithmetic, respectively. In addition, for algebra, the significant positive functional connectivity was observed between the visuospatial network (i.e., superior parietal lobule) and semantic network (i.e., inferior frontal gyrus and dorsomedial prefrontal cortex). For arithmetic, the significant positive functional connectivity was only observed between the visuospatial network (i.e., superior parietal lobule) and phonological network (i.e., precentral gyrus).

**Table 3:** Functional connectivity between seven ROIs for algebra and arithmetic

ROI to ROI	*t* value	*p* value (FDR-corrected)
Algebra		
IFG-AG	7.46	< .001
SPL-PCG	5.72	< .001
SPL-IFG	5.36	< .001
PCG-SMA	5.14	< .001
IFG-dmPFG	5.01	< .001
MTG-dmPFG	2.41	.029
AG-dmPFG	2.24	.029
SPL-dmPFG	2.16	.029
MTG-IFG	1.96	.045
Arithmetic		
IFG-AG	6.86	< .001
IFG-dmPFG	5.65	< .001
SPL-PCG	5.51	< .001
PCG-SMA	5.04	< .001
PCG-IFG	3.11	.004
MTG-AG	2.99	.011
MTG-IFG	2.89	.011
SMA-IFG	2.88	.011
AG-dmPFG	2.69	.012
MTG-dmPFG	2.49	.019

Semantic network: *MTG* middle temporal gyrus, *IFG* inferior frontal gyrus, dmPFC dorsomedial prefrontal cortex, *AG* angular gyrus. Phonologic network: *PCG* precentral gyrus, *SMA* supplementary motor area. Visuospatial network: *SPL* superior parietal lobule. Extent threshold: *p* < .05 FDR-corrected

## Discussion

The present study aimed to clarify the neural dissociation between algebra and arithmetic. Between-group brain activation analyses revealed that algebra resulted in greater activation in the bilateral angular gyrus, and arithmetic elicited greater activations in the bilateral supplementary motor area, left insula, and left inferior parietal lobule. Interindividual single-trial brain-behavior correlation analyses showed significantly positive brain-behavior correlations in the semantic network, including the bilateral middle temporal gyri, bilateral inferior frontal gyri, bilateral dorsomedial prefrontal cortices, and left angular gyrus for algebra. For arithmetic, the significantly negative brain-behavior correlations were observed in the phonological network, including the bilateral precentral gyrus, and left supplementary motor area, and in the visuospatial network, including the bilateral superior parietal lobules. For algebra, there was significantly positive functional connectivity between the visuospatial network and semantic network. For arithmetic, there was significantly positive functional connectivity between the visuospatial network and phonological network. These findings suggest that algebra relies more on the semantic network and arithmetic relies more on the phonological and visuospatial networks.

### Semantic network in algebra

The current study found that algebra relies more on the semantic network, including the bilateral angular gyrus, bilateral middle temporal gyri, bilateral inferior frontal gyri, bilateral dorsomedial prefrontal cortices, and left angular gyrus, than arithmetic. These regions overlap with the semantic network according to the meta-analysis of functional neuroimaging study by Binder et al. that was based on word processing [39]. Previous studies have revealed that the angular gyrus was critical for conceptual combination independent of the modality of the semantic content integrated [54–56], which indicated that the left angular gyrus was sensitive to compositional behavior of two-word phrases or tone sequences. For algebra processing, components including letters and operators were integrated to algebraic expressions similar to pure syntactic representation. The composition of operation and integration of algebra involved the conceptual combination. It elicited the activation the angular gyrus is to support the representation of integrated semantic information.

In algebra, formula derivations of the relationship between the letter and the operator (such as the commutative or associative laws) require knowledge of mathematical principles. Thus, algebra is similar to mathematical principles. Previous studies have shown that the mathematical principle processing based on conceptual knowledge is subserved by a semantic network [12, 40, 51, 57]. Liu et al. [51] reported that mathematical principles (e.g., exchanging the position of two operands in addition does not change their sum) elicited greater activation of the semantic network in the left middle temporal gyrus, left inferior frontal gyrus, and left angular gyrus than arithmetic computation (e.g., when the number 8 is first divided by 4 and then multiplied by 3, the final result is 6). The key role of the semantic network in mathematical principles suggests an important role of conceptual knowledge in algebra.

Furthermore, algebra requires abstract conceptual knowledge, and arithmetic involves concrete conceptual knowledge. Recent studies have suggested that concepts are organized in the brain according to semantic categories [58] such as animate versus inanimate [59] or concrete versus abstract [60] and that the classical semantic network is important for abstract conceptual knowledge [61–63]. A meta-analysis of studies regarding abstract concepts revealed that abstract concepts tend to produce stronger activation in the semantic network than concrete concepts [63]. By contrast, previous studies emphasized that abstract algebra concepts induced the non-linguistic cortical network dissociated from the classical semantic network [32, 33]. The subjective representation of the concreteness of algebra by expert mathematicians relied more on mental imagery, which differs from nonmathematical concepts in these studies [32, 33]. However, for laymen, algebra relies on the classical semantic network, suggesting a close association between algebra and language processing.

### Phonological and visuospatial networks in arithmetic

The present study found that arithmetic relied more on the phonological network, including the bilateral precentral gyri and supplementary motor areas, than algebra. It is hypothesized that arithmetic problems are solved by fact retrieval, in which the facts are stored as verbal codes [11, 24, 29], In this regard, arithmetic is related to phonological processing ability and elicits activity in the bilateral precentral gyri that supports phonological processing. On the other hand, arithmetic has greater demand on the phonological working memory, which supports the temporary storage and updating of intermediate results in the multi-step arithmetic [64]. These results are in line with models of arithmetic operations that outline a phonological processing network [25, 28, 65].

Our results also indicate that arithmetic results in greater activations in the visuospatial networks including the bilateral superior parietal lobules, which is consistent with the previously reported role of the visuospatial network in arithmetic [10–14, 28]. These regions are responsible for visuospatial information from the symbolic form of the arithmetic expression and reflect the visuospatial processing of concrete symbolic and quantitative representations in arithmetic. Numbers can elicit a visuospatial response, as the quantity of a number can be represented on a mental number line from left to right. This processing is evidenced by the spatial numerical association of response codes effect, which describes that people respond faster to small numbers with the left hand and large numbers with the right hand [66, 67]. Arithmetic can be completed using the visuospatial processing of a dynamic mental number line, as addition leads to a right shift and subtraction leads to a left shift in visuospatial processing [68–70].

### Significance of the single-trial brain-behavior correlation

Neuroimaging researchers often use interindividual brain-behavior correlations to explore the associations between the brain and human behaviors [71–73]. The correlation analysis investigates how interindividual differences in brain functions relate to interindividual behavioral performances and can help provide an accurate and representative neural mechanism of cognitive processing [49].

In this study, the traditional mean-trial brain-behavior correlations approach yielded correlations with *p* < 0.05, which is not considered significant in fMRI studies. The mean-trial correlation approach may not efficiently remove enough of the noise and does not result in a high correlation coefficient [74]. To improve the disadvantages of the traditional mean-trial correlation approach, we used the single-trial brain-behavior correlation approach previously reported in fMRI and ERP studies [42, 72]. The single-trial brain-behavior correlation approach avoids the influence of noise on the statistical analyses. This correlation approach first removes much of the noise (or residual activity) when determining the correlation for each trial and then conducts a *t*-test for the weak yet possibly stable correlation coefficients with the majority of the noise filtered. The results of this study showed significantly positive brain-behavior correlations in the semantic network for algebra and significantly negative brain-behavior correlations in the phonological and visuospatial networks for arithmetic (cluster-level *p* < 0.05 FDR-corrected with underlying voxel-level *p* < 0.001 uncorrected). In algebra processing, letters and operators were integrated to semantic concept combination. Brain activation increase in semantic network with longer response times because more neurons responding to conceptual combination are recruited for the successful performance of the task [75]. However, arithmetic processing relied more on procedural knowledge that was similar to automatic processing [76]. Brain activation decreases with longer response times because of more efficient use of brain pathways.

## Conclusion

Algebra is the abstract form of arithmetic and activates the semantic network, including the bilateral middle temporal gyri, bilateral inferior frontal gyri, bilateral dorsomedial prefrontal cortices, and left angular gyrus. In contrast, arithmetic activates the phonological network, including the bilateral precentral gyri and supplementary motor areas, and the visuospatial network, including the bilateral superior parietal lobules. These results suggest that the semantic network in the brain supports algebra. It deepened our understanding of the relationship between algebraic processing and arithmetic processing. Whereas previous studies suggested that algebraic processing activated the non-semantic cortical network in expert mathematicians [32, 33], our results highlighted the important role of the classical semantic network in algebraic processing. These findings have strong implications for education and teaching. In school-level education in general, algebra learning might take advantage of semantic knowledge.

## Data Availability

The data are currently not publicly available due to participant privacy, but they are available from the corresponding author upon reasonable request. Every request will be reviewed by the institutional review board of the State Key Laboratory of Cognitive Neuroscience and Learning at Beijing Normal University.

## Appendix

Materials used in the present study.

**Table:**

Algebraic task		Arithmetic task	
Expression 1	Expression 2	Expression 1	Expression 2
a−b−c	a−(b + c)	9–3−2	8−(1 + 3)
a ÷ b × c	(a × c) ÷ b	8 ÷ 4 × 3	(9 × 4) ÷ 6
a × (b + c)	a × b + a × c	2 × (6 + 2)	4 × 3 + 4 × 1
a ÷ b ÷ c	a ÷ (b × c)	8 ÷ 2 ÷ 2	6 ÷ (3 × 1)
a−b + c	a + (c−b)	9–6 + 3	8−(7−5)
a + (b + c)	(a + c) + b	7−(4 + 2)	(8−4)−3
a × (b × c)	(a × c) × b	8−(3 + 3)	9−(5 + 2)
a × b ÷ c	a × (b ÷ c)	(4 ÷ 2) × 2	8 ÷ (4 ÷ 2)
a-(b + c)	a ÷ b × c	8−(1 + 3)	8 ÷ 4 × 3
a × b + a × c	a−b + c	4 × 3 + 4 × 1	9−6 + 3
a × (b ÷ c)	a−b−c	(9 × 4) ÷ 6	(8−4)−3
a + (c−b)	a × (b + c)	8−(7−5)	2 × (6 + 2)
(a × c) × b	a ÷ b ÷ c	9−(5 + 2)	8 ÷ (4 ÷ 2)
a ÷ (b × c)	a × (b × c)	(4 ÷ 2) × 2	8−(3 + 3)
(a × c) ÷ b	(a + c) + b	8 ÷ 2 ÷ 2	9−3−2
a + (b + c)	a × b ÷ c	7−(4 + 2)	6 ÷ (3 × 1)

## Abbreviations

IPS: Intraparietal sulcus
fMRI: Functional magnetic resonance imaging
EPI: Echo planar imaging
ANOVA: Analysis of variance
ERP: Event-related potential
RTs: Reaction times
MNI: Montreal Neurological Institute
ROI: Region of interest
